# The Safety and Feasibility of a Family First Aid Approach for the Management of Postpartum Hemorrhage in Home Births: A Pre-post Intervention Study in Rural Pakistan

**DOI:** 10.1007/s10995-020-03047-6

**Published:** 2020-11-26

**Authors:** Meighan Mary, Sadiqua Jafarey, Rasha Dabash, Imtiaz Kamal, Arjumand Rabbani, Dina Abbas, Jill Durocher, Yi-Ling Tan, Beverly Winikoff

**Affiliations:** 1 Gynuity Health Projects, 220 East 42nd Street Suite 710, New York, NY 10017 USA; 2 National Committee for Maternal and Neonatal Health, Karachi, Pakistan

**Keywords:** Postpartum hemorrhage (PPH), Misoprostol, PPH management, Home births, Self-care interventions

## Abstract

**Objective:**

To evaluate the safety and feasibility of a Family First Aid approach whereby women and their families are provided misoprostol in advance to manage postpartum hemorrhage (PPH) in home births.

**Methods:**

A 12-month prospective, pre-post intervention study was conducted from February 2017 to February 2018. Women in their second and third trimesters were enrolled at home visits. Participants and their families received educational materials and were counseled on how to diagnose excessive bleeding and the importance of seeking care at a facility if PPH occurs. In the intervention phase, participants were also given misoprostol and counselled on how to administer the four 200 mcg tablets for first aid in case of PPH. Participants were followed-up postpartum to collect data on use of misoprostol for Family First Aid at home deliveries (primary outcome) and record maternal and perinatal outcomes.

**Results:**

Of the 4008 participants enrolled, 97% were successfully followed-up postpartum. Half of the participants in each phase delivered at home. Among home deliveries, the odds of reporting PPH almost doubled among in the intervention phase (OR 1.98; CI 1.43, 2.76). Among those reporting PPH, women in the intervention phase were significantly more likely to have received PPH treatment (OR 10.49; CI 3.37, 32.71) and 90% administered the dose correctly. No maternal deaths, invasive procedures or surgery were reported in either phase after home deliveries.

**Conclusions:**

The Family First Aid approach is a safe and feasible model of care that provides timely PPH treatment to women delivering at home in rural communities.

## Significance Statement

Misoprostol is effective in treating postpartum hemorrhage. Recent community-based studies have also confirmed that community health workers or birth attendants can diagnose and safely administer misoprostol for PPH management in home deliveries where timely referrals are complicated by a range of factors. This research demonstrates the safety and feasibility of delegating PPH diagnosis and treatment using misoprostol to women and their families. The study also demonstrates that innovative self-care approaches to improving access to PPH care within rural communities do not discourage institutional deliveries and, in fact, can promote community engagement and bolster the public health systems’ reach.

## Introduction

Globally, maternal mortality is on the decline, yet an estimated 300,000 maternal deaths occur annually and are mostly concentrated in low- and middle-income countries (WHO et al. 2015). The single largest cause of these deaths is postpartum hemorrhage (PPH), i.e. excessive bleeding after birth (Say et al. 2014). While efficacious interventions exist to help manage this condition, community-based strategies are urgently needed to help women who do not have access to skilled care at birth and may require immediate treatment to control postpartum bleeding. Misoprostol, a uterotonic pill, is a viable option in these situations, especially given international recommendations that support its use when the provision of intravenous oxytocin (the gold standard for treatment of atonic PPH) is not feasible (ICM and FIGO 2014; WHO 2012).

Based on misoprostol’s known efficacy in treating hemorrhage from two large trials conducted in hospital settings (Blum et al. 2010; Winikoff 2010), community-based studies have explored the potential of offering 800 mcg sublingual misoprostol for PPH treatment in home deliveries, administered by a community health worker or birth attendant prior to referral (Abbas et al. 2019). These studies confirmed that lay workers can safely administer misoprostol therapeutically and also highlighted the need to make treatment available, particularly in light of the social and cultural challenges around referrals that were documented for women diagnosed with PPH in remote settings (Abbas et al. 2019). Also, community-based research on use of misoprostol for prevention of PPH has documented rates of PPH from 6 to 16% despite receipt of prophylaxis (Abbas et al. 2019; Mobeen et al. 2011). These insights confirm that referral cannot be relied on as the sole solution for treatment for excessive postpartum bleeding following home birth (Bohren et al. 2014; Thaddeus and Maine 1994).

Strategies that rely on the provision of misoprostol to birth attendants or community health workers alone may not be sufficient to ensure access to timely care in every situation (Abbas et al. 2019). Approximately one-fourth of women globally deliver without skilled birth attendants (United Nations Children’s Fund 2018). Community strategies to facilitate early recognition of PPH and accessible treatment that can be administered by any birth attendant, companion, family member, or the woman herself are urgently needed. This community-based research sought to evaluate the safety and feasibility of a self-care model of PPH detection and management whereby women and their families were provided an 800 mcg dose of misoprostol in advance to use, if needed, as first aid for heavy bleeding following home births in rural Pakistan.

## Methods

A 12-month prospective, pre-post intervention study was conducted from February 2017 to February 2018 in eight rural union councils in the Badin and Mirpurkhas districts of Sindh Province, Pakistan to determine the feasibility and safety of the Family First Aid model: advance provision of misoprostol for use as a first aid measure to control excessive bleeding following home delivery. The eight union councils were selected due to high reported maternal mortality ratios (MMR: 345–350) and low frequency of skilled attendance at birth (49%) (UNDP Pakistan and Government of Sindh 2011). In these areas, standard of care for any woman with a complication during a home delivery was transfer to a health facility for emergency care (referral). The study was approved by the Ethical Review Committee of Ziauddin University, Karachi on November 12, 2016 in accordance with the 1964 Declaration of Helsinki.

For one month prior to the launch of the pre-intervention phase, trained data collectors conducted a district-wide mapping of pregnant women in the study area. Throughout the study, staff worked closely with community gatekeepers and traditional birth attendants (TBA). To facilitate identification of pregnant women and ensure retention and rapid follow-up of participants, home visits and regular check-in calls with local TBAs were implemented.

In both study phases, study staff visited pregnant women’s homes to confirm their estimated delivery date. Women were eligible if they were pregnant in their second or third trimester, resided in a study area, and agreed to consent. There were no exclusion criteria. Eligible women were informed about the research procedures before written consent was obtained. Oral consent was also an option for women who were not literate and was documented by a close family member, if present. Informed consent emphasized the voluntary and anonymous nature of the study; personal identifying information was not collected.

During the pre-intervention phase, participants received a counseling session (typically 45–60 min in length) including educational materials on birth preparedness, pregnancy danger signs, and newborn care at the enrollment visit. Along with their families, participants were also counseled on how to identify excessive bleeding and on the importance of seeking care at a facility if excessive bleeding occurred. Women received information on how to use traditional cloths, commonly placed underneath the woman at the time of delivery in this setting, to help estimate the quantity of blood loss. Women and their families were informed that two cloths soaked in blood or signs and symptoms of deteriorating health (profuse bleeding, paleness, difficulty in breathing, faintness) should indicate that the woman requires immediate attention and referral.

In the intervention phase, receipt of educational materials and counseling on how to identify heavy bleeding remained unchanged from the first study phase. In addition, participants received an advance dose of misoprostol (four 200 mcg tablets) at the enrollment visit to administer as first aid if PPH was suspected. Information and instructions on how to administer the pills sublingually, manage possible side effects, and the importance of seeking immediate medical care if bleeding continued following misoprostol administration was also provided.

During both study phases, background demographics and pregnancy characteristics were collected at the time of enrollment. Based on estimated delivery date, study staff contacted women and their families directly or were alerted by TBAs of recent deliveries to complete subsequent follow-up home visits within 1 week postpartum. At follow-up visits, data were collected on maternal and perinatal outcomes, and women were provided with a small gift of local dried fruit for their cooperation. During the intervention phase, follow-up data also included information on women’s experiences with misoprostol, including questions on reasons for use, knowledge about misoprostol, and acceptability of the Family First Aid model.

The primary outcome of the study was the proportion of women reporting use of misoprostol for Family First Aid for excessive bleeding at home deliveries. Home deliveries were defined as any delivery taking place within the community without skilled delivery attendance (e.g., woman’s home, TBA’s home, in transit within the community). Other key outcomes included: rates of PPH, rates of transfer to higher levels of care, and additional medical intervention and care received.

The pre-post intervention study was time-based in design and did not have a defined sample size. The pre-intervention phase occurred between February and July 2017 and the intervention phase from August 2017 to February 2018. Data were collected on all enrolled deliveries during each of the study phases. Based on available demographic data in the selected districts, we estimated that approximately 4330 women would deliver over the course of the year. With an anticipated 20% combined refusal and lost to follow-up rate, we projected that 3464 women (approximately 1723 in each phase) would be enrolled and followed-up postpartum.

Background and delivery characteristics were compared between study phases for all participants who completed follow-up visits postpartum. To determine if the Family First Aid model improved access to PPH care, analysis of key study outcomes by phase were conducted among women experiencing home deliveries, using chi square, Fisher’s exact, or t tests, as appropriate. Odds ratios (OR) with 95% confidence intervals were calculated to measure this effect. Descriptive sub-analyses of data collected during the intervention phase were conducted to evaluate the feasibility and acceptability of advance provision of misoprostol as part the Family First Aid model. All data were entered, cleaned, and analyzed using SPSS 21.0 software (IBM, Chicago, IL, USA).

## Results

**Fig. 1:**
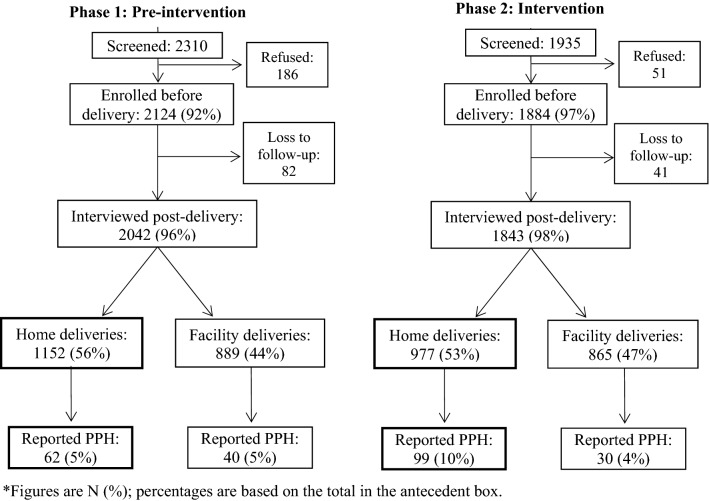
Participant flow

A total of 4245 eligible women were screened for participation in the study, of which 4008 were consented and enrolled. Approximately 97% of participants were successfully followed-up a median of 4 days after delivery; follow-up rates were similar between the two study phases. However, in the intervention phase, participants that were lost to follow-up (LTF) differed from retained participants on baseline education and parity; 12% of retained participants were educated compared to none in the LTF group (p = 0.01). The average number of live births among retained and LTF participants was 2.4 and 1.7 (p = 0.03), respectively. Approximately half of the deliveries followed-up in each phase took place in the community (pre-intervention: 1152; intervention: 977) (Fig. 1).

A comparison of participant and delivery characteristics between the pre-intervention and intervention study periods revealed some noteworthy differences in relation to care-seeking patterns. Compared to the pre-intervention phase, women who delivered during the intervention study period had a higher rate of attending 3+ antenatal care visits (pre-intervention: 17.8%, intervention: 32%, p < 0 .001) and higher reported use of iron folate during pregnancy (pre-intervention: 56%, intervention: 63.2%; p < 0 .001). Institutional delivery rates also significantly increased during the study (pre-intervention: 44%; intervention: 47% (p = 0 .04). While the overall rate of PPH, based on women’s reports, increased from 5% pre-intervention to 7% (p = 0 .01) during the intervention phase, only one maternal death was reported during the study—at a facility delivery during the pre-intervention phase. Overall reported perinatal mortality rates did not significantly change during the study (pre-intervention: 6.2%, intervention: 5.3%; p = 0 .85).

Among home deliveries, participant demographics were similar in the two study phases (Table 1). On average, women who delivered at home were 27 years old, with a reported gravidity of 4, and largely no formal education (pre-intervention: 93.9%; intervention: 92.5%). Participants’ use of services within the health system also significantly increased among this cohort. Women were significantly more likely to report having received antenatal care during the intervention phase; rates of women having attended at least three antenatal care visits increased from 10.9% in phase 1 to 20.6% in phase 2 (OR 2.13; CI 1.67, 2.71). Similarly among home deliveries, significantly more women reported taking iron folate during their pregnancy in phase 2 (OR 1.44; CI 1.22, 1.72). Home deliveries were most often attended by a TBA (98% in each phase).

**Table 1:** Participant delivery characteristics and outcomes by study phase among home deliveries % (n) or m (± sd)

	Pre-intervention phase (n = 1152)	Intervention phase (n = 977)
Age, mean^a^	27.4 (4.8)	27.1 (4.8)
Any form of education	6.1% (70)	7.5% (73)
Parity, mean	2.67 (2.17)	2.61 (2.3)
3+ antenatal care visits***	10.9 (125)	20.6 (201)
Iron folate taken during pregnancy***	43.3 (498)	52.5 (513)
Delivery assistance^c^		
No one	0.4 (5)	0.2 (2)
Skilled provider	0.7 (8)	0.6 (6)
CHW	0.2 (2)	0.1 (1)
TBA***	98.3 (1132)	98.4 (958)
Quack ***	6.4 (74)	1.3 (13)
Family member***	5.3 (61)	21.5 (209)
Non-technical pharmacist***	3.5 (40)	12 (117)
Reported PPH	5.4 (62)	10.1 (99)
Maternal deaths	0	0
Perinatal deaths^d^	4.3 (49)	3.8 (37)

^a^Excluding unknown responses: 617 in pre-intervention phase and 704 in intervention phase

^b^Home deliveries include woman’s home, TBA’s home and in-transit (< 0.5%)

^c^Multiple responses chosen; excluding unknown responses: 3 in intervention phase

^d^Among singletons only; pre-intervention n = 1146 intervention n = 974

***p <  0.001

The odds of reporting excessive bleeding almost doubled (pre-intervention: 5.4%; intervention: 10.1%; OR 1.98; CI 1.43, 2.76). Among participants that reported excessive bleeding (Table 2), women in the Family First Aid intervention phase were over ten times more likely to have received some treatment for PPH (OR 10.49; CI 3.37, 32.71), compared to women in pre-intervention study period. Most of these women (93%) used misoprostol as first aid treatment. Significantly fewer participants reported receiving care for excessive bleeding from a quack or local dispenser during the intervention phase (10%), compared to 44% in the first study phase (p <  0.001). During the intervention phase, significantly fewer referrals occurred following home deliveries (1%), compared to the pre-intervention phase (14.5%) when misoprostol was not available to women (OR 0.06; CI 0.01, 0.49). No invasive procedures or surgery were reported in either phase after home deliveries—nor were there any maternal deaths.

**Table 2:** PPH management and health outcomes among reported PPH cases at home deliveries % (n)

	Pre-intervention phase (n = 62)	Intervention phase (n = 99)
Reported care received for PPH		
None***	30.6 (19)	1.0 (1)
From TBA/unskilled provider**	11.3 (7)	1.0 (1)
From skilled provider**	14.5 (9)	1.0 (1)
From quack or dispenser***	43.5 (27)	10.1 (10)
Family First Aid (misoprostol)	n/a	92.9 (92)
PPH cases referred for care at a health facility***	14.5 (9)	1.0 (1)
Care received at referral health facilities^a^		
Uterotonics (oxytocin)	77.8 (7)	100 (1)
Tranexamic acid	11.1 (1)	0
IV fluids	100 (9)	100 (1)
Bimanual compression	11.1 (1)	0
Uterine packing	33.3 (3)	0
Manual removal of placenta	11.1 (1)	0
Analgesia	88.9 (8)	100 (1)
Transfusion	33.3 (3)	0

^a^Pre-intervention phase n = 9; intervention phase n = 1

***p <  0.001, **p <  0.01

Among women who reported taking misoprostol for excessive postpartum bleeding, 90% administered the misoprostol dose correctly (following the study regimen): all women reported using the sublingual route of administration, 91% reported taking all four tablets, and 99% of women took the misoprostol dose within 24 h after expulsion of the placenta (Table 3). In all instances, misoprostol was used after the birth of the baby in response to bleeding. Some women reported difficulties (9.8%) with taking misoprostol due its unappealing taste and side effects. Almost all women (98%) reported being “satisfied” or “very satisfied” with using misoprostol as a first aid measure to control heavy bleeding after childbirth within their community. Sub analysis of women in the intervention phase indicated that women almost universally (> 99%) understood, why, when, and how to take misoprostol for PPH treatment in the Family First Aid model, irrespective of delivery location or actual use of the medicine (Table 4).

**Table 3:** Reported use of misoprostol for Family First Aid among reported PPH cases at home deliveries % (n)

	Intervention phase (n = 99)
Administered misoprostol for family first aid	92.9 (92)
Reason for taking misoprostol^a^	
Excessive blood loss identified	87 (80)
To prevent bleeding after childbirth	14.3 (14)
Advised by family or TBA	1.0 (1)
For safety	3.1 (3)
Assistance in taking misoprostol^a^	
No one	32.6 (30)
TBA	30.4 (28)
Family member	44.6 (41)
Quack doctor	2.2 (2)
Timing of misoprostol administration^a^	
Within an hour after delivery (after expulsion of placenta)	94.6 (87)
1–24 h after delivery	4.3 (4)
More than 24 h after delivery	1.1 (1)
Misoprostol dose administered^a^	
1–3 tablets	8.7 (7)
4 tablets	91.3 (84)
Route of administration^a^	
Sublingual	100 (92)
Correct administration (timing, dose, route)^a^	90.2 (83)

^a^n = 92

**Table 4:** Reported knowledge of misoprostol for Family First Aid among participants that delivered at home % (n)

	Intervention phase (n = 977)
Purpose of misoprostol	
To treat excessive bleeding after childbirth	99 (967)
To prevent bleeding after childbirth	0.7 (7)
Other	0.1 (1)
Don’t know	0.2 (2)
When to take misoprostol	
Always immediately after the delivery of the baby	0.4 (5)
Only if she experiences excessive bleeding after delivery	99.2 (969)
Don’t know	0.4 (4)
Route of administration	
Sublingual	99.4 (971)
Oral	0.1 (1)
Don’t know	0.5 (5)
Dose of misoprostol	
1–3 tablets	0.7 (7)
4 tablets	99.1 (968)
Don’t know	0.2 (2)
What to do if bleeding continues after misoprostol administration	
Go to facility	99.7 (974)
Other	0.1 (1)
Don’t know	0.2 (2)

## Discussion

The Family First Aid approach evaluated in this study provides a unique model for ensuring immediate access to PPH care for women in even the most remote regions who may be less likely to receive timely care, if any. By empowering women delivering at home and their companions to be engaged in postpartum monitoring, our study showed that the Family First Aid approach is a safe and feasible model of self-care that provides effective treatment to women with excessive bleeding within rural communities.

A review of global experiences on the implementation of advance distribution models of misoprostol for PPH prevention reveals similar findings that support the safety and feasibility of self-use of misoprostol for prophylaxis (Smith et al. 2016). The provision of misoprostol for both prevention and treatment is another community-based approach that has been investigated, though it requires even more resources (Abbas et al. 2019). Based on the evidence, it is unclear if universal prevention of PPH using misoprostol, plus treatment with misoprostol as needed, is more effective in preventing poor outcomes than early recognition and treatment of PPH as a more targeted prevention strategy in community settings. Studies in India comparing the use of a 600 mcg oral dose of misoprostol prophylaxis to an early treatment regimen of 800 mcg sublingual misoprostol for women who bled more than 350 mL demonstrated that early treatment is non-inferior and more cost effective than universal prophylaxis (Chatterjee et al. 2016; Raghavan et al. 2016). These findings suggest that a Family First Aid model may also be a more feasible alternative to promoting a universal prophylaxis approach, which helps reduce blood loss but still lacks evidence on its impact on mortality (Hobday et al.).

The Family First Aid model does not replace the critical need for medical care for the small percentage of women with PPH refractory to initial misoprostol treatment. However, such cases are relatively rare (< 1%) as documented in this study and in other studies (Blum et al. 2010; Winikoff 2010). More importantly, for women unable to reach a health facility in a timely manner or at all, this model increases access to PPH treatment and engages families and birth attendants in the active monitoring of women during and after delivery.

Placing pills in women’s hands may increase PPH diagnosis. Women without access to misoprostol may be less likely to report PPH unless it’s a severe case since there is no readily available treatment. Alternatively, when women have the ability to take tangible action by administrating misoprostol, they may be more inclined to report PPH and administer the medicine provided, a possible explanation for the increased rate of reported PPH in the intervention group. Yet, given the simplicity and safety of misoprostol administration, an increase in PPH diagnosis and misoprostol use is preferable to the risks of morbidity and mortality related to delayed PPH care. Also, provision of misoprostol directly to women and families may reduce potential risks associated with use of medicines and supplies from other sources (i.e. quacks and local dispensers) where drug quality is not regulated and cannot be assured in this setting.

While misoprostol is increasingly available and registered around the world for PPH management, many local pharmacies do not have misoprostol in stock. Supply chain issues need to be considered when assessing the feasibility of scale-up of a universal community distribution model such as the Family First Aid approach. Furthermore, community distribution models are highly dependent on the functionality of health systems and have been shown to be complicated to sustain at scale in fragile health systems (Hobday et al. 2018; Rajbhandari et al. 2017; Smith et al. 2016). The cost-effectiveness of the Family First Aid approach also merits further inquiry in this context. While a misoprostol treatment dose only costs approximately 0.24USD in Pakistan, an economic cost–benefit analysis is necessary to assess scalability of the Family First Aid approach which provides all pregnant women with misoprostol treatment when drugs may only be needed by approximately 10% of women (those who have PPH). Nonetheless, the Family First Aid approach warrants further consideration where women face considerable barriers to reaching referral facilities due to geographic, socio-cultural, or economic challenges.

High rates of reported TBA-assisted home deliveries in this study suggest that an alternative mechanism of increasing access to Family First Aid and/or timely PPH care could be implemented by training and equipping TBAs as opposed to distributing misoprostol to women directly. TBAs are currently not provided supplies nor recognized as skilled birth attendants in Pakistan; but if trained, they could work with families to identify heavy bleeding and administer PPH treatment using misoprostol. TBAs in many settings, including in Pakistan, have been sidelined from maternal health strategies and programs that are largely focused on facility births and “skilled” providers (Hobday et al. 2018; Prata et al. 2011). Given shortages in skilled personnel and continual challenges in providing good quality care for facility births (Montagu et al. 2017), many women continue to rely on community TBAs, in addition to family and other unskilled companions at home births. The findings of this research suggest that TBA’s equipped with misoprostol as first aid providers could be a viable option to address the leading cause of maternal mortality in rural communities.

Introduction of educational materials reinforcing safe pregnancy and delivery practices during both study phases may have contributed to the significant increases observed in the proportion of women seeking antenatal care and institutional delivery. Since no other program was introduced in the selected union councils during the study period to affect health service uptake, community awareness associated with continual study staff presence is also likely to have had a favorable impact on these outcomes. These study findings confirm established evidence that community-level PPH management models do not discourage women from delivering at health facilities or engaging with health systems (Geller et al. 2014; Sanghvi et al. 2010; Smith et al. 2014). Providing and empowering women with information and commodities may actually improve confidence in and linkages to the formal health sector as families feel supported in their choice of where to deliver and when to seek care.

While this study provides important insight into an innovative Family First Aid approach and the possible role of self-care in PPH management to reach more women who would otherwise have little or no access to care, it does have limitations. As with many pre-post intervention studies, the sample was too small to assess the effectiveness of this approach on morbidity, mortality, and other rare secondary outcomes (i.e., rates of transfer to higher levels of care, need for additional medical intervention and care, etc.). Since postpartum follow-up was conducted at each participant’s household, recall bias and error is possible in the self-reporting of PPH care received. In addition, study activities were conducted by hired staff rather than by community health workers or lay providers affiliated with the public health system to reflect actual programmatic implementation.

Further, while this study was conducted in a rural setting and may not be generalizable to other contexts, the results documented in this very low resource context suggests a need to rethink how women and families can be more integrated into improving care in all delivery locations, including institutional deliveries. Evidence from observational studies (Oladapo et al. 2007) suggests that even women who deliver in facilities experience delays in PPH diagnosis due to lack of monitoring, personnel shortages, and uterotonic stock outs. The value of birth companions in improving delivery outcomes is well documented (Bohren et al. 2017) and current WHO recommendations promote offering all women the choice of birth companions before and during labor (Tunçalp et al. 2015; WHO 2018a). Our study findings also highlight the important role they might play in postpartum monitoring during a critical period when the majority of maternal deaths occur (Nour 2008). Engaging birth companions in postpartum monitoring for complications such as PPH may improve health outcomes at both home and facility births. Future research should explore the impact of task-shifting simple first aid PPH measures, including postpartum monitoring, PPH diagnosis, and misoprostol administration to a range of birth companions, no matter where the woman delivers, in efforts to reduce delays and bottlenecks in timely PPH care.

## Conclusions

Despite increasing evidence emphasizing the safety and feasibility of prophylactic misoprostol administration by women and birth companions, current international guidelines still limit the use of uterotonics to trained health workers (WHO 2018b). This research contributes to and expands the current evidence base by demonstrating the safety and feasibility of delegating PPH recognition and first aid management to women and their families using an innovative self-care approach. The study also demonstrates that community-based approaches can improve access to timely PPH care within rural communities without demotivating women from opting to deliver at a facility. In fact, this model of care may bolster community confidence in health systems while expanding the reach of simple PPH solutions to women, no matter where they decide to deliver.
